# Recent developments in non-coplanar radiotherapy

**DOI:** 10.1259/bjr.20180908

**Published:** 2019-02-01

**Authors:** Gregory Smyth, Philip M Evans, Jeffrey C Bamber, James L Bedford

**Affiliations:** 1 Joint Department of Physics, The Institute of Cancer Research and The Royal Marsden NHS Foundation Trust, London, UK; 2 Centre for Vision Speech and Signal Processing, University of Surrey, Guildford, UK; 3 National Physical Laboratory, Hampton Road, Teddington, Middlesex, UK

## Abstract

This paper gives an overview of recent developments in non-coplanar intensity modulated radiotherapy (IMRT) and volumetric modulated arc therapy (VMAT). Modern linear accelerators are capable of automating motion around multiple axes, allowing efficient delivery of highly non-coplanar radiotherapy techniques. Novel techniques developed for C-arm and non-standard linac geometries, methods of optimization, and clinical applications are reviewed. The additional degrees of freedom are shown to increase the therapeutic ratio, either through dose escalation to the target or dose reduction to functionally important organs at risk, by multiple research groups. Although significant work is still needed to translate these new non-coplanar radiotherapy techniques into the clinic, clinical implementation should be prioritized. Recent developments in non-coplanar radiotherapy demonstrate that it continues to have a place in modern cancer treatment.

## Introduction

Non-coplanar radiotherapy uses a number of fixed or rotating radiation beams that do not share the same geometric plane relative to the patient.^1^ This reduces the beam overlap away from the tumour. Conventional C-arm linear accelerators (linacs) achieve this by rotating the recumbent patient around the isocentre on a treatment couch to a different position for each beam orientation.^1^ Non-coplanar radiotherapy is more common in intracranial stereotactic radiotherapy, single-fraction radiosurgery (SRS, Table 1) and stereotactic body radiotherapy (SBRT).^1^ These techniques often deliver higher fractional doses and require highly conformal, sharp dose gradients outside the planning target volume (PTV) to minimize dose to adjacent normal tissue.^2^ Non-coplanar beams are also used in accelerated partial breast irradiation (APBI) to spare the ipsilateral breast,^3^ which may improve cosmetic outcomes.^4^ In head and neck cancer non-coplanar radiotherapy reduces the low and intermediate dose bath,^5^ which may decrease the incidence of neurocognitive side-effects and fatigue.^6,7^

**Table 1.** A list of acronyms used throughout this review

**Acronym**	**Complete form**
APBI	Accelerated partial breast irradiation
BOO	Beam orientation optimization
DWA	Dynamic wave arc
IMRT	Intensity modulated radiotherapy
NC-IMRT	Non-coplanar intensity modulated radiotherapy
NC-VMAT	Non-coplanar volumetric modulated arc therapy
OAR	Organ at risk
PTV	Planning target volume
SCNC-VMAT	Static couch non-coplanar volumetric modulated arc therapy
SRS	Stereotactic radiosurgery
SBRT	Stereotactic body radiotherapy
TSP	Travelling salesman problem
VMAT	Volumetric modulated arc therapy

The need for manual intervention to rotate the patient couch makes non-coplanar radiotherapy time-consuming when using C-arm linacs. The adoption of volumetric modulated arc therapy (VMAT), an efficient rotational intensity modulated radiotherapy (IMRT) delivery technique,^8–11^ also makes non-coplanar beam arrangements less appealing in practice. However, recently there has been renewed interest in non-coplanar radiotherapy, as modern linacs allow automated motion of multiple rotation axes.^12^

This review aims to give an overview of recent developments in non-coplanar radiotherapy. We aim to answer three questions: (1) Which modern non-coplanar radiotherapy techniques have been developed (Table 2) and what sites might benefit from their use? (2) What technological and computational approaches are required for treatment planning and delivery? (3) What issues must be resolved prior to the clinical implementation of new non-coplanar radiotherapy techniques?

**Table 2.** A summary of the non-coplanar radiotherapy techniques discussed in this review

**Technique**	**Linac geometry**	**Non-coplanar geometry achieved by**	**Key references**
Non-coplanar intensity modulated radiotherapy	C-arm linac	Multiple static beams defined by linac gantry rotation and patient couch rotation	^13–22^
Static couch non-coplanar volumetric modulated arc therapy	C-arm linac	One or more arcs, some with a non-zero patient couch rotation	^23–27^
Coronal VMAT	C-arm linac	One or more arcs achieved with dynamic patient couch rotation but with fixed or limited linac gantry rotation. Trajectories may be manually defined, calculated or optimized.	^28–31^
Trajectory VMAT	C-arm linac	One or more arcs with dynamic patient couch rotation and dynamic linac gantry rotation. Trajectories may be manually defined, calculated or optimized.	^32–44^
CyberArc	Robotic arm mounted linac	One or more arcs defined by robotic arm orientation.	^45,46^
Dynamic Wave Arc	O-ring linac	One or more arcs with dynamic linac gantry rotation around the horizontal and vertical axes. Trajectories may be manually defined, calculated or optimized.	^47–50^

VMAT, volumetric arc therapy.

## Recent developments in non-coplanar IMRT

### Non-coplanar IMRT for C-arm linacs

Non-coplanar IMRT (NC-IMRT) has been generally limited to a small number of beam orientations due to the increased delivery time required. However, with modern automated delivery, the use of NC-IMRT with 20 or more beams may now be practical.^12^ Research in this area is led by a group at the University of California, Los Angeles (UCLA).

The group’s initial work has focused on SBRT for liver^13^ and lung^14^ tumours, where dose escalation is technically challenging due to the proximity of critical organs. In both cases, organ at risk (OAR) constraints prevent dose escalation for complex cases, or require compromises in tumour dose to avoid unacceptable risk of toxicity. For liver SBRT, 14- and 22-beam NC-IMRT plans are compared with coplanar VMAT and are found to reduce normal liver dose.^13^ For lung SBRT, 30-beam NC-IMRT is compared with VMAT and enables dose escalation to the tumour by an additional 20 Gy while conventional OAR dose constraints are still met.^14^ Alternatively, OAR doses can be reduced while delivering the conventional prescription dose to the tumour.

Subsequent work has investigated other sites where dose escalation is thought likely to be beneficial or is technically feasible if OAR doses can be maintained.^15,16^ In glioblastoma, critical structures such as the brainstem often abut or overlap the PTV, which limits the prescription dose to 60 Gy. Nguyen et al investigate the potential of dose escalation of the PTV, the GTV alone, and treating an expanded PTV using 30-beam NC-IMRT.^15^ Although dose escalation up to 100 Gy is feasible, using such a high prescription in practice is questionable due to the increased risk of brain necrosis above 60 Gy.^51^ Dose escalation for SBRT in head and neck cancer patients by up to 20 Gy is also technically possible using 30-beam NC-IMRT.^16^ However, in practice, care is required when the tumour lies close to critical structures, in this case the carotid artery.

These studies provide the initial evidence that by employing a large number of beams, NC-IMRT produces highly conformal dose distributions that can further reduce OAR doses, dose-escalate the tumour while observing OAR dose constraints, or make treatment of challenging body sites practical. Additional treatment planning studies by this group investigate NC-IMRT for cancers of the prostate,^17,18^ liver,^19^ and brain.^20^

**Figure 1.**
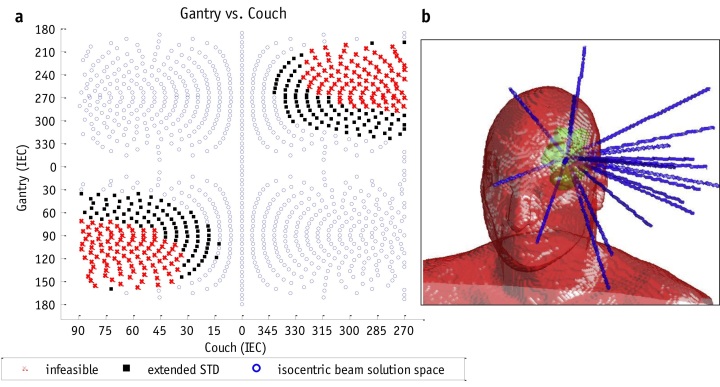
The geometry for non-coplanar intensity modulated radiotherapy demonstrating (a) the feasible non-collisional search space for non-coplanar beam orientation defined by gantry and couch rotation angles (°), and (b) the final optimized beam orientations for a clinical patient plan. STD, source to target distance; IEC, International Electrotechnical Commission. Reprinted from Yu et al,^21^ with permission from Elsevier.

NC-IMRT has been clinically implemented within a prospective Phase I trial for patients requiring retreatment of primary brain tumours.^21^ Patients who have been previously treated to 59.4 or 60 Gy receive a further 25 or 30 Gy in 5 or 10 fractions. Plans using 13–20 beams (median = 16) are compared with static couch non-coplanar VMAT. Plans are judged on the basis of PTV coverage and OAR sparing, and the preferred plan is treated. Of the 10 patients in the study whose plans meet acceptable OAR tolerances, 9 have been treated with NC-IMRT and 1 patient has been treated with a VMAT plan of equivalent quality. The NC-IMRT beam orientation search space and the beam arrangement for an example case are shown in Figure 1.

### Optimization techniques for non-coplanar IMRT

Determining the optimal set of beam orientations for a clinical case is challenging. As plan quality does not vary smoothly with changes in beam orientation, the solution space is likely to contain local optima.^52^ Many groups have investigated beam orientation optimization (BOO) for IMRT, and the literature has been extensively reviewed previously.^53,54^ Only the application of these BOO methods to new non-coplanar radiotherapy techniques is covered in this paper.

NC-IMRT work that has been reported by the UCLA group uses an iterative approach to BOO.^13^ Iterative BOO uses fluence optimization to evaluate plan quality during BOO^55^ and has been applied to a wide range of clinical cases by the ERASMUS group in Rotterdam.^56–64^ Although fluence optimization does not account for the effects of practical machine delivery constraints present in a clinical treatment plan, the idealized dose distribution it produces can give a useful estimate of plan quality. New orientations are added to a beam arrangement until either the maximum permitted number of beams is reached, or the effect of adding another beam no longer significantly improves the optimization objective function. However, the scheme is slow to converge^65^ and can be trapped in a local minimum by the first beam chosen.^56^

In the UCLA implementation of iterative BOO, at each iteration the beam orientation that most reduces the objective function is added. The objective function improvement for each potential beam is estimated using a single iteration of fluence optimization, which results in a more efficient search.^13^

## Recent developments in non-coplanar VMAT

### Non-coplanar VMAT for C-arm mounted linacs

**Figure 2.**
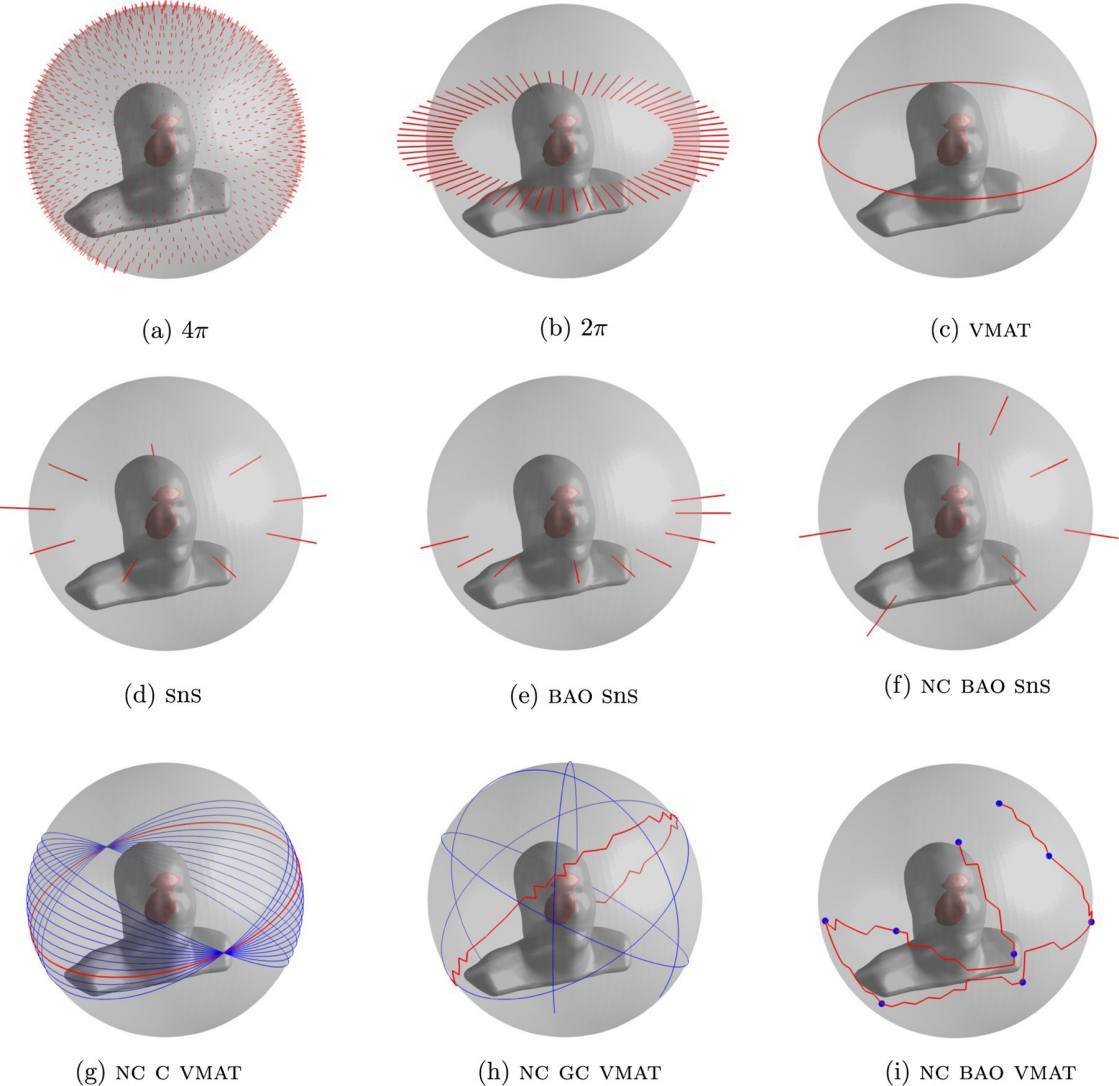
Available treatment geometries for coplanar and non-coplanar radiotherapy. An upper limit on treatment plan quality can be determined by distributing a large number of beams over the full (a) non-coplanar or (b) coplanar space. Other techniques shown are: (c) coplanar VMAT, (d) coplanar IMRT, (e) coplanar IMRT with optimized beam orientations, (f) non-coplanar IMRT with optimized beam orientations, (g) static couch non-coplanar VMAT, (h) non-coplanar trajectory VMAT tracing the great circles around the patient, and (i), non-coplanar trajectory VMAT visiting nine optimized beam orientations. BAO, beam angle optimized, equivalent to BOO in this review; IMRT, intensity modulated radiotherapy; SnS, step and shoot, a type of IMRT delivery; VMAT, volumetric arc therapy. Reprinted from Wild et al^32^ with permission from John Wiley and Sons, ^©^ American Association of Physicists in Medicine.

Several papers have proposed methods of non-coplanar VMAT treatment delivery. These break down into three areas: (1) VMAT with multiple static couch rotations, (2) a coronal VMAT technique that combines dynamic couch rotation with fixed gantry positions, and (3) a trajectory VMAT technique that combines dynamic couch rotation with dynamic gantry rotation. Feasible orientations for non-coplanar VMAT, as well as a range of other techniques, are shown in Figure 2.

#### Static couch non-coplanar VMAT

The simplest application of non-coplanar radiotherapy to VMAT uses one or more arcs with static couch rotations. Although it has been investigated for sites such as sinus cancer,^66^ liver,^22^ and head and neck,^32,63^ it is commonly used for intracranial stereotactic radiotherapy and SRS.^23,24^

Four-arc static couch non-coplanar VMAT (SCNC-VMAT) improves conformity and reduces the volume of brain receiving intermediate doses in twelve single-lesion SRS cases, when compared with coplanar VMAT and nine-field NC-IMRT.^23^ However, the best technique for sparing OARs close to the PTV depends on the patient’s specific geometry. An alternative SCNC-VMAT technique, which combines three non-coplanar arcs and one coplanar arc, has been evaluated for up to nine lesions.^24,25^ This class solution has since been incorporated into the Eclipse (Varian Medical Systems, Palo Alto, CA) treatment planning system as HyperArc.^26^

HyperArc combines SCNC-VMAT with standardized immobilization devices, to prevent collisions, and automated transitions between each partial arc during treatment, to improve delivery efficiency. HyperArc reduces dose to normal brain tissue when compared against VMAT for 23 SRS cases, with up to 4 lesions each.^27^ However, beams are more complex and require more monitor units due to increased modulation. Another study of 15 SRS cases, with between 3 and 8 lesions each, does not find significant differences between HyperArc, CyberKnife (Accuray Inc, Sunnyvale, CA) and VMAT for most OAR criteria studied.^26^ Differences in homogeneity and the volume of tissue receiving 110% of the prescription dose are significant between HyperArc and CyberKnife but this may be due to different planning approaches across software.

#### Coronal VMAT

Dynamic couch rotation with fixed lateral gantry positions, to achieve a coronal VMAT technique, has been proposed for APBI.^28^ Treatment planning for this site aims to deliver a homogeneous dose to the partial breast PTV, while minimizing the dose to other OARs, including the heart, lungs, and contralateral breast. When coronal VMAT is combined with up to 20^◦^ of manually defined gantry rotation, ipsilateral lung dose is reduced at the expense of increased ipsilateral breast dose in patients with inner and central tumours compared with coplanar VMAT.^29^

**Figure 3.**
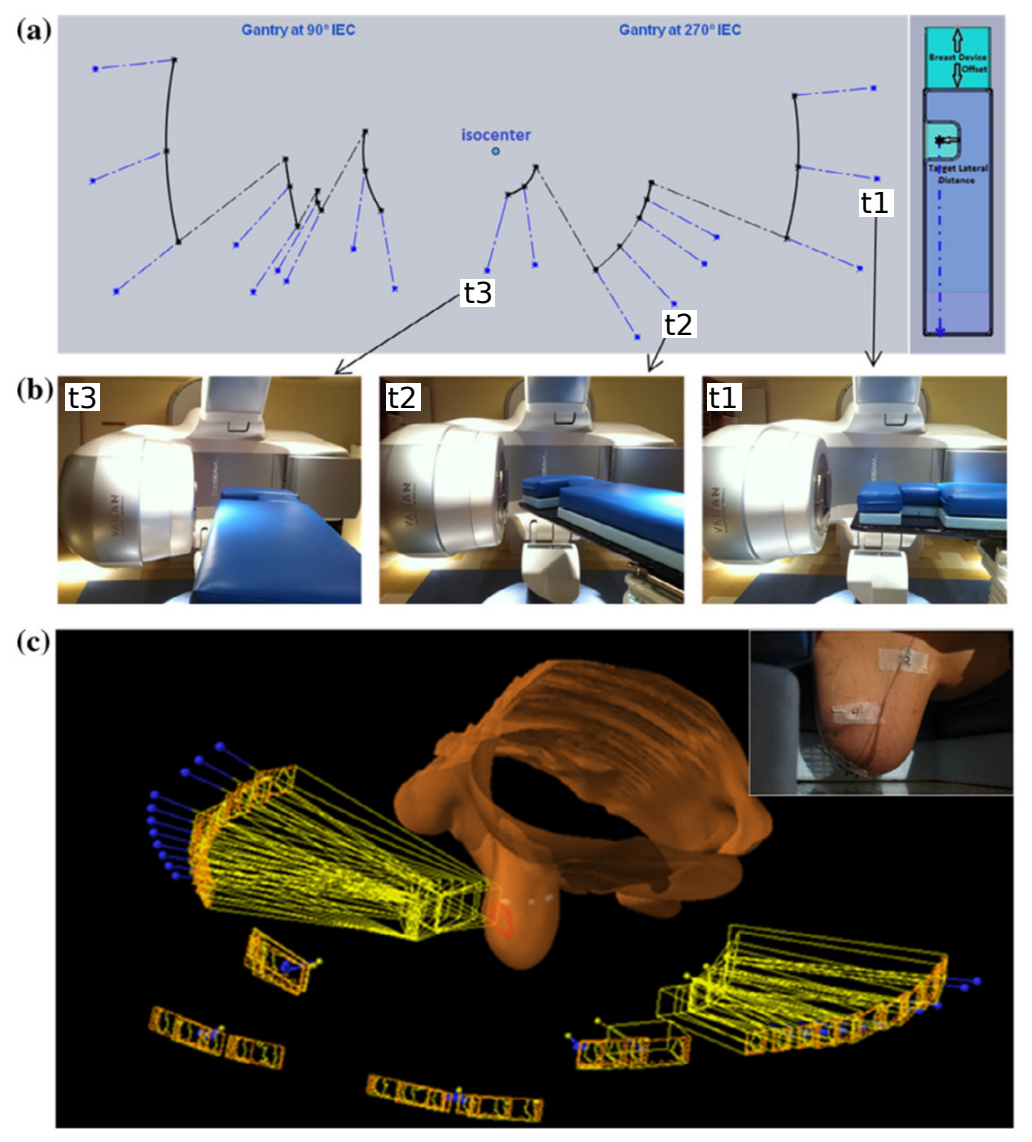
A coronal VMAT technique demonstrating (a) a discontinuous non-isocentric trajectory, (b) the linac orientations corresponding to points on the trajectory, and (c) the three-dimensional view of the beam and treatment geometry. Reprinted from Fahimian et al,^30^ with permission from Elsevier. VMAT, volumetric arc therapy.

Coronal VMAT has been refined for prone patient orientations (Figure 3), using lateral couch translations to avoid collisions between the linac gantry and patient couch.^30,31^ This produces a discontinuous, non-isocentric beam trajectory. Coronal VMAT improves conformity and reduces the volume of the ipsilateral normal breast receiving high and intermediate doses, when compared with six-field NC-IMRT for 10 cases, although the volume of low dose (V20%) increases.^31^ However, patients with unfavourable PTV locations have been excluded from the study, suggesting that coronal VMAT has limitations for specific geometries.

#### Trajectory VMAT

Combining dynamic couch rotation with conventional gantry rotation to produce a trajectory VMAT technique may be more promising than coronal VMAT due to the additional degree of freedom. Originally proposed for brain and head and neck cancers,^33,34^ several groups have demonstrated the use of different patient-specific trajectory VMAT techniques for OAR sparing. These include: multiple partial arcs,^35^ trajectories approximated by SCNC-VMAT,^36^ multiple partial arc rotations of the gantry for a single continuous couch rotation,^37^ a single continuous rotation of the gantry with synchronised couch rotation,^38,39^ or a single continuous rotation of the couch with synchronised gantry rotation.^40^

Due to the additional non-collisional space superiorly, compared with other treatment sites, brain cancer is commonly investigated. Trajectory VMAT shows significant OAR sparing compared to coplanar VMAT for multiple optimization techniques.^35,36,38–42^ OAR sparing results depend on the inputs to the trajectory and plan optimization, however, it is possible to produce clinically significant sparing for structures such as the contralateral hippocampus and temporal lobe.^39^

Other sites that have been investigated include: head and neck,^32^ lung,^40,41^ prostate,^38,40^ and liver.^42^ However, small numbers of cases are used to validate individual optimization algorithms. More extensive investigations are required to determine if any dosimetric improvements for these sites are present over patient populations and are clinically significant.

### Optimization techniques for non-coplanar VMAT

#### Manual and algorithmic methods

Manual trajectory definition is used in the earliest work on non-coplanar arc techniques, where beam overlap within the patient from different arc sectors is minimized.^33^ At that time, linac modifications had been required to enable the continuous dynamic couch rotation, so it has not been widely used.^37^ This approach has been revisited recently, using a manually defined sinusoidal pattern with up to nine partial gantry rotations.^37^ Although this form of trajectory VMAT improves conformity over simpler non-coplanar conformal arcs, it is dosimetrically equivalent to SCNC-VMAT.

Trajectory definition is also used for coronal VMAT, with an algorithm that maximises couch rotation while ensuring the PTV lies within the limits of the beam’s eye view.^30,31^ Collisions between linac components are avoided by a combination of modelling and lateral couch translations. Once the trajectory is determined, VMAT optimization is performed to define the final beam apertures.

The common factor for manual and algorithmic techniques is that there is no direct method of trajectory optimization. However, these relatively simple methods to avoid OARs or to smear out the low dose within the patient have been shown to improve dosimetry for specific applications.

#### Beam scoring methods

Beam scoring methods, which evaluate a quality metric for each feasible beam orientation, are frequently used for IMRT beam orientation optimization (BOO). Their advantage is speed, as scoring a single beam orientation is fast and the many separate evaluations needed can be performed in parallel.^67^ Most beam scoring techniques separate BOO from treatment plan optimization, which further reduces complexity. However, evaluating beams independently may not identify a beam arrangement that produces the optimal treatment plan.

Trajectory optimization employing beam scoring generally uses the patient’s geometry to determine individual beam scores, either projecting it onto the beam’s eye view plane^35,36^ or after ray tracing.^38,39^ Beam scoring has been refined to reflect the relative clinical importance of OARs,^36,39^ OAR position relative to the PTV,^36,43^ and to incorporate dosimetric information for individual voxels.^67^ After each feasible beam orientation has been scored, the trajectory is determined from high-quality orientations. Published techniques include: grouping promising orientations into partial arcs,^35^ reducing the path to a series of fixed couch positions,^36^ and determining a single connected trajectory.^38–40,43^ This last approach casts trajectory optimization as a path-finding problem, which is solved using graph-search techniques such as the Dijkstra^38–40^ or A*^42,43^ algorithms. However, the result also depends on the rules permitted for trajectory formation and this approach would not find any higher quality multiple partial-arc trajectories.

#### Fluence-based methods

Although beam scoring produces high quality treatment plans, the final trajectories may not be dosimetrically optimal as plan quality is not directly evaluated during trajectory optimization. An alternative approach incorporates fluence optimization into trajectory optimization, as a measure of plan quality.

Fluence-based BOO techniques have been applied to trajectory optimization by initially solving a static field IMRT BOO problem and using the resulting beam orientations to define a limited number of angular positions that must be visited during delivery.^32,41^ To create the final optimized trajectory, these orientations must be linked together in some way.

One method of connecting these orientations is to formulate a travelling salesman problem (TSP) to determine the most efficient trajectory that visits all the selected beams. Although the IMRT beams chosen during BOO are of high quality, this may not be true of the linking sections. These could degrade plan quality by including a section of trajectory that disproportionately irradiates OARs compared to the PTV. However, this may not be a significant factor in practice, as the MLC apertures and dose contribution are determined subsequently during plan optimization and would compensate for poor choices of trajectory sections resulting from the TSP.^32^

To avoid this problem, an alternative approach replaces the treatment efficiency metric in the TSP with a separate beam scoring approach. By using beam scores, the TSP can then be solved using an A* path-finding algorithm. High-quality connections between optimal beam orientations are then determined and infeasible sections of arc are also avoided.^42^

Alternative fluence-based techniques attempt to evaluate the quality of the whole trajectory during optimization, rather than basing the trajectory on a small number of optimized beam orientations. Dong et al investigate Monte Carlo Tree Search, which performs fluence optimization on selected trajectories and uses the results as feedback to guide the selection of promising trajectories in later iterations.^44^ Another approach alternates between BOO and trajectory formation until a final trajectory is found.^40^ Fluence-based VMAT optimization is performed using a technique that encourages a sparse solution of promising beam orientations. The results from this optimization then define the inputs for a trajectory optimization step, which is formulated as a graph-search problem using fluence information and solved with Dijkstra’s algorithm.

Dosimetric information can be incorporated into trajectory optimization by perturbing an initial trajectory based on a fluence optimization.^39^ This allows alternative solutions to be investigated as changes to anchor points along an input trajectory are iteratively tested. The input trajectory can be either a coplanar arc or the output of another trajectory optimization algorithm.

### Non-coplanar VMAT for O-ring mounted linacs

The VERO (Mitsubishi Heavy Industries, Tokyo, Japan and Brainlab AG, Feldkirchen, Germany) O-ring mounted linac can deliver a trajectory VMAT technique by rotating around the vertical axis.^68^ Dynamic Wave Arc (DWA) has been shown to produce equivalent or better OAR sparing compared to coplanar VMAT for a number of clinical sites^47,48^ and has been clinically implemented in at least one centre.^49^ Published studies use manually defined trajectories for treatment plan optimization within the iPlan (Brainlab AG, Feldkirchen, Germany) or RayStation (RaySearch, Stockholm) systems.^49,50^ However, as dynamic couch rotation for C-arm linacs and dynamic ring rotation for O-ring mounted linacs are equivalent from the patient’s point of view, the optimization techniques described above can be adapted for the VERO system.

## Recent developments for CyberKnife

The CyberKnife system is a robotic arm-mounted linac, which delivers multiple non-coplanar, non-isocentric beams from a set of pre-defined beam orientations.^69–71^ It is frequently used for intracranial stereotactic radiotherapy, SRS and SBRT, and for retreatments. However, treatment times can be up to 1 h in duration, including patient positioning and imaging.^45^

Delivery times can be significantly improved while maintaining treatment plan quality by optimizing the selection of a limited number of beams from those available.^72^ Alternatively, an arc optimization scheme has been proposed for CyberKnife (CyberArc) that uses a similar approach to that employed in VMAT techniques. It has been developed for treatments using a variable Iris collimator^45^ and has been adapted for use with the CyberKnife multileaf collimator.^46^ As the CyberKnife treatment planning system can already produce high quality NC-IMRT plans, the arc optimization attempts to match the dose distribution from a clinically acceptable static beam plan but to produce a more efficient delivery. By allowing continuous radiation delivery between nodes in an optimized trajectory, estimated delivery times are between one-third and half of the original treatment plan.

## Challenges and barriers to clinical implementation

### Delivery efficiency

Delivery of non-coplanar treatment plans can be time consuming, which could limit the clinical implementation of novel techniques. For the nine patients that have been treated using NC-IMRT within a Phase I trial, the average delivery time is 34.1 min (range 19.9–64.5 min) for 16 (13-20) beams and 5 Gy (3–6 Gy) per fraction.^21^ However, motion of the machine axes between beams is the major component of the delivery times and this could be significantly reduced with fully automated machine transitions between beams. Coronal VMAT delivery is between 4.5 and 5 min for a 3.85 Gy fraction partial breast treatment.^30,31^ Trajectory VMAT is delivered in around 2 min for a 1.8 Gy fraction brain treatment^73^ and 3–8 min for 12–15 Gy SRS.^37^ Although these delivery investigations use a non-clinical research mode, the results demonstrate the potential efficiency gains with fully automated delivery.

### Delivery accuracy

Coplanar VMAT requires accurate synchronization of MLC motion, gantry rotation, and dose rate.^74^ For NC-VMAT, additional synchronization of these components with patient couch rotation is required.^75^ The dosimetric accuracy of NC-VMAT has been investigated for coronal VMAT,^30,31^ as well as mathematically defined^37^ and geometrically optimized^73^ trajectory VMAT. For all techniques, absolute point dose measurements are within 3% and at least 90% of film pixels report a γ value <1 for 3% and 3 mm criteria.^30,31,37,43,73^ These results suggest that, with a fine control point spacing for all motion axes, NC-VMAT is sufficiently accurate for clinical use.

### Patient safety and compliance

Automated delivery of NC-IMRT or NC-VMAT risks collisions between linac and patient support systems or with the patient themselves. The main concern is for patient safety during delivery, primarily in avoiding collisions of the linac with the patient. However, this is challenging as potential collisions are patient, treatment site and immobilization device-dependent. Identifying a collision when the patient is on the treatment couch is not sufficient, as creating a new plan with adjusted trajectories would have a significant impact on clinical resources and patient scheduling. Therefore, current machine interlocks such as touch-guards or imaging-based collision detection, while still necessary, are insufficient on their own. Unless pre-defined trajectories and approved immobilization devices are used, such as for HyperArc, advanced patient modelling and collision prediction techniques must be incorporated into the planning process prior to trajectory optimization.^76^ Perceptions of collision risk could also affect patient compliance, however compliance for NC-IMRT of brain tumour retreatment was found to be good.^21^

### Intrafraction patient motion

Intrafraction patient motion for non-coplanar radiotherapy has two potential causes. Firstly, the change in position of the anatomy during the treatment fraction, which may increase with any extension of treatment duration for non-coplanar techniques. Secondly, any change in position of the anatomy that is induced by the novel delivery techniques described above, *e.g.* during automated motion of the treatment couch. Intrafraction motion has been quantified within a trial of NC-IMRT for intracranial tumours and is within 1 mm for all but one case (1.5 mm).^21^ However, intrafraction motion must be investigated for other body sites and the need for additional immobilization for dynamic couch techniques should also be determined.

Alternative linac configurations pose fewer problems for intrafraction motion. The O-ring mounted linac of the VERO machine avoids concerns around patient-linac collisions, additional immobilization or intrafraction motion with DWA. However, the achievable range of non-coplanar orientations is restricted due to the potential for collisions between the couch and O-ring,^50^ which may limit its use for intracranial sites. Intrafraction motion for the CyberKnife is less problematic due to its imaging and tracking system.^71^ Applying similar monitoring and intrafraction motion prediction modelling may assist the introduction of non-coplanar trajectories within the clinic.

## Summary and conclusions

Recent developments in non-coplanar radiotherapy show that it continues to have a place in modern cancer treatment, particularly for intracranial sites, stereotactic radiotherapy, or in cases of retreatment. A substantial body of work has investigated novel methods of delivering and optimizing non-coplanar radiotherapy (Table 3). The potential of extra degrees of freedom to increase the therapeutic ratio, either through dose escalation to the target or dose reduction to functionally important organs at risk, has been demonstrated by multiple research groups. Although significant work is still needed to translate these new non-coplanar radiotherapy techniques into the clinic, particularly to ensure patient safety, clinical implementation should be prioritised within the remit of a clinical trial.

**Table 3.** Summary of the applications, optimization methods, and readiness for clinical implementation of the non-coplanar radiotherapy techniques discussed in this review

**Technique**	**Clinical sites investigated**	**Technological approach**	**Computational methods**	**Clinical implementation**	**Challenges**
NC-IMRT	Liver^13,19,22^ lung^14^ brain^15,20,21^ head & neck^16^ prostate^17,18^	Up to 30 static non-coplanar beams	Beam orientation optimization using existing methods^13–22^	Ready for implementation	Automated trajectory sequencing
SCNC-VMAT	Brain^23–27^	VMAT with multiple fixed patient couch rotations	Manual selection from limited arc set	Ready for implementation	Automated delivery and collision prevention on non-HyperArc platforms
Coronal VMAT	Partial breast^28–31^	Dynamic patient couch rotation with fixed or limited linac gantry rotation	Manual^28,29^ or algorithmic^30,31^ trajectory definition	Requires substantial further development	Collision prevention Intrafraction motion Patient compliance Investigation of other clinical sites Non-research delivery technology
Trajectory VMAT	Brain^33,35–42,44^ head & neck^32,34,43^ prostate^38,40,44^ lung^40–42,44^ liver^42^ chest wall^44^ oesophagus^44^	Synchronized dynamic patient couch rotation and linac gantry rotation.	Manual^33,34^ or mathematical^37^ trajectory definition Beam scoring^35,36,38,39^ or fluence-based^32,39–42,44^ trajectory optimization	Requires substantial further development	Collision prevention Intrafraction motion Patient compliance Non-research delivery technology
CyberArc	Brain and prostate^45,46^	Arc delivery sequencing for robotic arm mounted linac	Dose mimicking and fluence-based trajectory optimization^45,46^	Requires some further development	Integration into proprietary treatment planning and linac control software
Dynamic Wave Arc	Brain^48^ metastatic disease^47,49^ prostate^47,49^ pancreas^47^ lung^47,49^ breast^49^	Dynamic rotation of O-ring linac	Manual definition^47–50^	Ready for implementation	Application of trajectory optimization techniques to O-ring linac geometry

IMRT, intensity modulated radiotherapy; VMAT, volumetric arc therapy.

The techniques and their abbreviations are defined in Table 2
